# Orodispersible Films with Rupatadine Fumarate Enclosed in Ethylcellulose Microparticles as Drug Delivery Platform with Taste-Masking Effect

**DOI:** 10.3390/ma15062126

**Published:** 2022-03-14

**Authors:** Katarzyna Olechno, Bartosz Maciejewski, Klaudia Głowacz, Joanna Lenik, Patrycja Ciosek-Skibińska, Anna Basa, Katarzyna Winnicka

**Affiliations:** 1 Department of Pharmaceutical Technology, Medical University of Bialystok, Mickiewicza 2c, 15-222 Bialystok, Poland; 2 Department of Pharmaceutical Technology, Medical University of Gdansk, Hallera 107, 80-416 Gdansk, Poland; bartosz.maciejewski@gumed.edu.pl; 3 Chair of Medical Biotechnology, Warsaw University of Technology, Noakowskiego 3, 00-664 Warsaw, Poland; kglowacz@ch.pw.edu.pl (K.G.); pciosek@ch.pw.edu.pl (P.C.-S.); 4 Department of Analytical Chemistry, Institute of Chemical Sciences, Faculty of Chemistry, Maria Curie-Skłodowska University, M. Curie-Skłodowska Sq. 3, 20-031 Lublin, Poland; joanna.lenik@mail.umcs.pl; 5 Institute of Chemistry, University of Bialystok, Ciolkowskiego 1k, 15-245 Bialystok, Poland; abasa@uwb.edu.pl

**Keywords:** orodispersible film, ethylcellulose, microparticles, spray drying, rupatadine fumarate, taste-masking, polymeric materials

## Abstract

Orally disintegrating (orodispersible) films provide a versatile tool for drug administration, especially in the pediatric and geriatric population, since they reduce the risk of choking and do not necessitate drinking water during application. By considering their direct contact with the taste buds, palatability is an influential aspect related to patient compliance. The microparticles based on taste-masking polymers containing drugs enclosed inside effectively mask the unpleasant taste of medicines. Ethylcellulose is a hydrophobic polymer widely used as a taste-masking material. Rupatadine fumarate, a second-generation antihistamine drug, is characterised by an intense bitter taste; therefore, it is crucial to achieve a tolerable taste whilst developing orodispersible formulations with its content. The objective of this study was to develop orally disintegrating films with rupatadine fumarate in the form of ethylcellulose-based microparticles obtained from aqueous dispersions of ethylcellulose—Surelease^®^ or Aquacoat^®^ ECD. It was a technological challenge to achieve homogenous drug content per dosage unit and sufficient mechanical properties for film operating due to the necessity to suspend the microparticles in the casting solution. Although the process of obtaining films consisted of several steps (mixing, pouring, drying), the particles were homogeneously dispersed, and each film of the desired size contained the proper dose of the drug. The taste-masking effect was also maintained. This parameter was confirmed by three independent methods: in vivo by healthy volunteers, an electronic tongue and a dissolution test. The applied taste-evaluation techniques showed that the films containing Aquacoat^®^ ECD microparticles have the highest degree of bitter taste reduction, which confirms the results obtained in our previous studies.

## 1. Introduction

A common issue in pediatric as well as ageing patients is swallowing difficulty influencing the acceptance of solid drug dosage forms; therefore, making the need to generate age-appropriate medicines imperative. In order to surmount the swallowing complication, carers frequently manipulate the medicines—they split/crush tablets or open capsules and mix their contents with food or liquid. The administration of medication in liquid form also involves problems of measuring the correct dose and swallowing the specified volume. Auspicious and individualised drug delivery systems for these patients constitute oral polymeric strips, known as orodispersible films (ODFs) [1,2,3,4]. ODFs are characterised by high ease of application; the precision of drug dosing and do not require liquid sipping during administration. These features are particularly beneficial in the treatment of pediatric, geriatric and bedridden patients, as well as psychiatric patients who simulate taking medication, as the film dissolves faster than they are able to spit it out. Overall, ODFs help patients who have an aversion to swallowing a tablet or capsule and those who find it extremely difficult to take standard tablets because of dysphagia or other problems with swallowing (e.g., the elderly and infirm) [4,5,6,7]. It is also worth mentioning that ODFs as a drug dosage form is recommended to be used in children already from 6 months of age [3,4,8]. 

ODFs constitute modern, solid drug dosage forms, which appear as thin, polymeric strips with a thickness of 12 to 100 μm and an area of 2 to 8 cm^2^ [5,6]. They are intended for oral administration, where they disintegrate quickly in contact with saliva, thus eliminating the risk of choking. The European Pharmacopoeia (Ph. Eur.) does not specify the disintegration time for ODFs; however, it provides it for other orodispersible forms —tablets (orally disintegrating tablets, ODT), which is a maximum of 3 min and was also adopted for films. In turn, the Food and Drug Administration (FDA) indicates a maximum disintegration time of 30 s for ODT, which is far more appropriate for the targeted patient use and also reflects the desired disintegration time for ODFs [6,9,10,11].

Excipients are the most important part of ODFs, as they ensure good sensory properties and adequate mechanical performance of the formulation. The primary excipients in designing ODFs are film-forming polymers, which form their structure, and plasticisers, which are responsible for the flexibility of the film and prevent it from crumbling. The polymers typically used in designing ODFs are hydrophilic cellulose derivatives: methylcellulose, carboxymethylcellulose, hydroxypropylcellulose and hydroxypropylmethylcellulose (HPMC); and polyvinyl alcohol, sodium alginate, gelatin, chitosan or pullulan. Among them, HPMC is widely and often utilised, especially because of its safety after oral administration and the possibility of use in children. The most commonly used plasticisers are glycerol, polyethyleneglycols, sorbitol and mannitol. It is notable that plasticisers have a sweet, pleasant taste; therefore, these excipients simultaneously act as a substance influencing taste sensations. Other excipients include flavour masking agents, surfactants or saliva stimulants. It should be emphasised that the most important feature of all excipients used in film technology is their inertness on the mucous membrane, non-toxicity and non-irritation after application. The production of films is carried out by using the following methods: solvent casting, hot-melt extrusion and electrospinning [12,13,14]. 

Palatability is one of the essential elements in the acceptance of a drug product, which is determined as the general perception of medicine according to organoleptic attributes such as odor, taste, aftertaste and texture (mouthfeel). Due to the direct contact of ODFs with taste buds, masking the unpleasant taste of the active substance is an important factor influencing the efficiency of the pharmacotherapy process. It should be emphasised that children are especially sensitive to bitterness [3,4]. Preparing microparticles (MPs) using taste-masking polymers is considered to be an efficient method for reducing the bitterness of a drug [15,16,17]. One of the hydrophobic polymers applied as a taste-masking agent is ethylcellulose (EC), which is a biocompatible and nonirritant polymer. EC is considered a safe excipient permitted for use in drugs (included in the FDA’s database of excipients). According to the World Health Organization (WHO) Food additives Series, EC has low oral toxicity. Importantly, the polymer is being used in pediatric and parenteral formulations. Due to the fact that EC is water-insoluble while soluble only in organic solvents, it creates an effective barrier against the movement of drug molecules to the surface and water molecules to the core, providing a taste-masking effect. Commercially, EC is available in powder form (e.g., Aqualon or Ethoce) or aqueous dispersion (Surelease^®^, Aquacoat^®^ ECD, Aquarius™ Control ECD and AshaKote) [17,18,19,20].

Rupatadine fumarate (RUP) is one of the newest antihistamines with a multidirectional mechanism of action, used both in child (above two years old) and adult therapy. It is a potent, selective histamine receptor and platelet-activating factor (PAF) receptor antagonist, which specifically distinguishes RUP from other drugs in this group and explains its innovative mechanism of anti-inflammatory action. This feature is clinically relevant to PAF-induced allergic inflammatory processes and symptoms of bronchial hyperreactivity [21,22]

The purpose of the present study was to design ODFs containing incorporated MPs with RUP. The technological problem connected with the development of this dosage form was to provide adequate pharmaceutical properties and to simultaneously maintain the taste-masking effect of bitter RUP. In our former study, MPs formulated with different forms of EC were analysed for taste-masking potential. Those obtained from an aqueous dispersion of EC (Surelease^®^, Aquacoat^®^ ECD) were considered to be advantageous in terms of taste-masking efficacy and morphology [17], and they were utilised for ODFs development. ODFs were assessed for morphological structure, mass and thickness uniformity, mechanical properties, drug content and potential interactions. Disintegration time was assessed in vivo in healthy volunteers, in Petri dish and by drop method. The key assessment of taste-masking efficiency was performed by three different approaches: in vivo, based on a release test, and by using an electronic tongue (an array of sensors exhibiting cross-sensitivity coupled with data analysis techniques).

## 2. Materials and Methods

### 2.1. Materials

Aquacoat^®^ ECD was given from FMC BioPolymer, Newark, NJ, USA. Surelease^®^ E-7-19040 was donated from Colorcon Inc., Harleysville, PA, USA. Hypromellose (HPMC) Pharmacoat^®^ 606 was handed over from Shin-Etsu Chemical Co., Ltd., Tokyo, Japan. Glycerol was procured from Fagron sp. z o.o., Kraków, Poland. RUP was purchased from Xi’An Kerui Biotechnology Co., Ltd., Xi’An, China.

### 2.2. Preparation of MPs and ODFs

MPs were obtained by the spray drying method using a Mini Spray Dryer B-290 (Büchi, Switzerland) equipped with a standard 0.7 mm nozzle, with the utilisation of Surelease^®^ (MP-S-RUP) or Aquacoat^®^ ECD (MP-A-RUP) (selected during preliminary studies EC aqueous dispersions) diluted with water to an appropriate concentration, and combined with RUP. Previous research performed by our team showed that MPs based on the aqueous dispersions (Surelease^®^, Aquacoat^®^ ECD) were characterised by the highest degree of bitterness reduction [17]. The effective taste-masking barrier was created with RUP:polymer ratio (0.5:1) with 6% EC concentration; therefore, such compositions were used in the development of the ODFs. Placebo MPs were also prepared as a comparative control trial—MP-S and MP-A.

The spray drying process parameters were empirically determined as follows: inlet temperature 85 °C, outlet temperature 70 °C, aspirator flow 98% and flow rate 3.5 mL/min. All experiments were conducted under ambient conditions (20–25 °C, 40–50% RH) [17]. Under these process parameters, the process yield of 76.2% and 81.4% and encapsulation efficiency of 86.1% and 95.1% for MP-S-RUP and MP-A-RUP, respectively, were achieved. The mean size of MP-S-RUP was 3.2 ± 1.1 µm, and MP-A-RUP was 3.6 ± 0.5 µm [17].

ODFs were prepared by solvent casting method from a solution made of HPMC (in a concentration of 12%), glycerol (50% *w/w* of the polymer) and purified water. The concentration values were established experimentally. The polymer was dissolved in water containing a plasticiser, and the obtained solution was homogenised using a mechanical stirrer. Then the appropriate MPs (MP-S-RUP or MP-A-RUP) or pure drugs in a precisely determined quantity were added, and the formulation was mixed. Four casting solutions were prepared (Table 1): placebo (F1), with pure, unprocessed RUP (F2), MP-S-RUP (F3) and MP-A-RUP (F4), and cast on the surface of 400 cm^2^ utilising an automatic film applicator (*Elcometer* 4340, Elcometer Limited, Manchester, Great Britan) equipped with a special adapter with casting height of 400 µm at speed 1 mm/sec. The casting surface of the applicator was heated to a temperature of 40 °C, which allowed for sufficiently fast evaporation of the solvent without affecting the properties of the emerging film. It was established that at temperatures above 50 °C, the films were brittle, while the temperature below 40 °C prolonged the drying time, which negatively affected the quality of microparticles.

The obtained films were cut into pieces (2 cm × 2 cm) to include 2.5 mg of RUP in each ODF, as 2.5 mg is the dose required for children weighing from 10 to 25 kg [23].

### 2.3. Viscosity Measurements

The viscosity of the solutions screened for the potential use in the manufacturing of ODFs was determined with a rotational viscometer Viscotester 6 Plus (Thermo Haake, Karlsruhe, Germany). Samples were subjected to a constant shear rate for 60 s. All measurements (*n* = 3) were conducted under ambient conditions (20–25 °C, 40–50% RH).

### 2.4. Evaluation of Morphology of ODFs

ODFs surface analysis was performed by scanning electron microscope (SEM, Inspect™S50, FEI Company, Hillsboro, OR, USA). Scraps of ODFs were placed on adhesive carbon tapes attached to the surface of a dedicated holder and covered in gold. Tests were performed at room temperature, using various magnifications.

### 2.5. Quality Assessment of ODFs

#### 2.5.1. Uniformity of Weight and Thickness

ODFs were weighted individually employing analytical balance (Radwag, Radom, Poland). The thickness was measured using a calibrated digital caliper (Beta 1651DGT, Milan, Italy) at five points of each ODF (two upper corners, middle and two lower corners) [24]. All measurements were performed under ambient conditions (20–25 °C, 40–50% relative humidity). The arithmetic mean and standard deviation (SD) of 3 samples were calculated for evaluation.

#### 2.5.2. Moisture Content

Content of moisture in single ODFs was assessed using moisture analyser balance (Radwag WSP 50SX, Radom, Poland). 

#### 2.5.3. Drug Content

Quantitative measurement of RUP for individual ODF was carried out using HPLC apparatus (Agilent Technologies 1200) featuring with Waters Spherisorb^®^ 5 μm ODS1 4.6 mm × 250 mm column (Waters Corporation, Milford, CT, USA). As a mobile phase methanol:phosphate buffer, pH = 3.0 (35:65, v/v) was utilised (isocratic elution). Flux was established as 1.0 mL/min and wavelength as 245 nm [25,26,27]. A buffer consisting of NaH_2_PO_4_ and water, adjusted to pH = 3.0 by H_3_PO_4_, was applied. The standard calibration curve was linear in the range of 1–100 μg/mL, and the correlation coefficient r was 0.999. The studies were carried out in triplicate.

#### 2.5.4. Mechanical Properties

Mechanical properties were generally assessed by the tensile strength (TS), Young’s modulus (E), percent of elongation at break (EB) and tear resistance (TR). While evaluating TS, the sample is stretched until it tears, and the stress needed represents the tensile strength. It is calculated by dividing the maximum force (N) needed for tearing the film (value of TR) by the cross-sectional area (mm^2^) of the film. Young’s modulus (elastic modulus, E) is the measure of the stiffness of the film—its value describes material resistance to deformation and might be designated by the slope on the stress/strain curve. The values of those parameters were analysed with a texture analyser (TA.XT plus, Stable Micro Systems, Godalming, UK) for 4 cm^2^ films with experimental parameters based on the tension test mode. A single film was attached in brackets, positioned at an original height of 20 mm. The samples were extended with a speed of 1 mm/s, with a cell loading of 5 kg [28,29,30].

The elasticity of oral films is an essential physical property required for simple application. The elasticity of ODF can be quantified by its folding endurance. The parameter was evaluated by repeatedly folding the film at 180° relative to a plane in the same plane until it breaks or folding up to 300 times without breaking. The number of times the films were folded without breaking was computed as the folding strength value [28,30]. 

All measurements were performed under ambient conditions (20–25 °C, 40−50% relative humidity). The arithmetic mean and standard deviation (SD) of 3 samples were calculated for evaluation.

### 2.6. Disintegration Time Assessment—In Vivo in Healthy Volunteers, Petri Dish, Drop Method

The disintegration time of ODFs in the oral cavity was evaluated by six healthy volunteers (Bioethics Commission Approval no. APK.002.112.2021). Individual ODF was held in the mouth without chewing until disintegrated and spitted out. Then, the mouth was rinsed with purified water. The time required for the complete disintegration in the oral cavity was noted. The test on a Petri dish was carried out with phosphate buffer (pH 6.8) at 37 °C [17], in an amount of 3 mL, mimicking the amount of saliva in the mouth, under gentle shaking. The disintegration time of the sample was taken when the test film started breaking as observed visually. The disintegration time was evaluated using the drop method as follows: a single film was placed onto a watch glass, and a single drop (0.2 mL) of phosphate buffer (pH 6.8) was dripped on the film surface; the time until medium penetrated through the film was recorded [24,28].

### 2.7. Differential Scanning Calorimetry (DSC)

Thermal analysis of pure, unprocessed RUP; HPMC; the physical mixture of HPMC and RUP; MPs placebo (MP-S, MP-A); MPs containing drugs, MP-S-RUP and MP-A-RUP; and fragments of films, F1, F2, F3 and F4, were accessed utilising thermal analyser system (DSC Mettler Toledo, Greifensee, Switzerland) at temperatures ranging from 40 °C to 300 °C with 10 °C/min rate with 20 mL/min flow of nitrogen. All samples were analysed in aluminum pans.

### 2.8. Evaluation of Taste-Masking Effectiveness

#### 2.8.1. In Vivo in Healthy Volunteers

Assessment of taste-masking effectiveness was evaluated in vivo in a human taste panel by six healthy volunteers (Bioethics Commission Approval no. APK.002.112.2021). The study was carried out in the following stages: single ODF emplaced on a tongue for 30 s, spitting out, mouth rinsing with water [17].

#### 2.8.2. RUP Dissolution

RUP dissolution was carried out by using mini paddle apparatus equipped with enhancer cells (Erweka Dissolution Tester DT 600HH, Heusenstamm, Germany) [31] immersed in 40 mL of phosphate buffer (pH 6.8), maintaining the following conditions: 75 rpm and 37 °C (+/−0.5). Individual ODF was placed in a single enhancer cell and covered with an ultra-thin cellulose membrane. At specified time periods, 1 mL samples were withdrawn, filtered and analysed. The quantity of dissolved RUP was assessed as pointed out in 2.5.3. Three repetitions for each one were performed.

#### 2.8.3. Electronic Tongue 

##### Membrane Materials 

For the preparation of polymer membrane, different groups of ingredients were utilised—polymer: poly(vinylchloride) (PVC) (Tarwinyl, Zakłady Azotowe w Tarnowie-Mościcach S.A., Tarnów-Mościce, Poland); plasticisers: o-nitrophenyl octyl ether (o-NPOE), bis(2-ethylhexyl) sebacate (DOS), (Fluka, St. Gallen, Switzerland); lipophilic salts: potassium tetrakis(p-chlorophenyl)borate (KTpCPB) (Fluka, St. Gallen, Switzerland), tetradodecylammonium tetrakis(4-chlorophenyl) borate (TDMA-TCPB); tetradodecylammonium chloride (TDAC), tridodecylmetylammonium nitrate (TDMAN) (Sigma-Aldrich, St. Luis, MO, USA); and ionophore: calix[6]arene-hexaacetic acid hexaethylester (amine ionophore I) (Fluka, St. Gallen, Switzerland). All aqueous solutions were prepared with deionised water of conductivity 0.07 μs/cm (Elix Advantage System Mili-Q plus Milipore, Spittal an der Drau, Austria). 

##### Fabrication of the Electrodes and Membrane Preparation 

The body of the electrodes was made from insulating plastic material. In the body, a cable was placed in which the silver–silver chloride electrode was soldered. The Teflon sensor was 1.5 cm long and 5 mm in diameter and connected with the body by the screw thread. The membrane phase of the electrode was 3 mm in diameter and consisted of two layers placed in a Teflon holder: the inner layer containing plasticised PVC in which the Ag/AgCl electrode was placed and the outer layer, contacting with the tested solution and containing the active substance apart from the inner layer components. The internal layer consisted following ingredients: 30% w/w PVC, 70% w/w of plasticisers, DOS or o-NPOE. The components were mixed, and the mixture was deaerated. The Teflon holders were filled with the mixture so that the silver–silver chloride electrode was immersed in it. In the next step, the mixture was gelated at 373 K for 30 min. The preparation of the outer layer membrane consists of weighing the inner layer components (Table 2), dissolving the obtaining mixture in tetrahydrofuran (THF), placing it in drops on the inner layer (several times), leaving at 293 K to evaporate THF and gelating the layer. The electrodes prepared were stored in the air between the measurements. 

##### Electronic Tongue Measurements and Performance of the Electrodes

The sensor array consisted of 16 ion-selective electrodes (two sensors of each type) with plasticised PVC membranes of solid contact architecture. The electrochemical cell can be represented as follows: Ag, AgCl; KCl 3M│CH3COOLi 1M│sample solution║membrane║PVC+plasticiser (solid contact); AgCl, Ag. The measurements of the electromotive force of the system ion-selective electrodes—reference electrode (Orion 90-02 Thermo Electron Corporation, Beverly, MA, USA)—were carried out using Electrochemistry EMF Interface system (Lawson Labs. Inc., Malvern, USA) and IBM PC computer. Before the first measurement, the electrodes were preconditioned for at least 3 days in the RUP solution of concentration 10^−3^ mol/L. Regarding the poor solubility of RUP in water, the calibration curves of the constructed electrodes were examined by measuring the EMFs in the concentration range of RUP 10^−6^–10^−3^ mol/L (for three repetitions). The calibration curves of tested electrodes in the solution of HPMC (0.4 g/L–0.004 g/L), MP-S-RUP and MP-A-RUP of concentration of 0.13 g/L–0.0013 g/L were determined. The sensitivity of the electrodes towards excipients was determined in the concentration range relevant to the performed release measurements. 

The used procedure for electronic tongue analysis is a standard measurement protocol applied previously for various pharmaceutical samples by our team [32]. It was applied to test taste-masking efficiency and release of drugs from the studied formulations. It consists of a few steps: signal stabilisation for 5 min (sensors immersed in deionised water), the introduction of the studied pharmaceutical formulation to the medium (t = 0) and recording of electrodes’ signals that are influenced by the released RUP and excipients, in time. The signals of the sensors were registered during 15 min (5 min stabilisation, 10 min release) in six independent replicates for each sample type. Between sample measurements, the sensors were washed with purified water and dried. Chemical images of investigated samples were obtained on the basis of signals of 16 potentiometric sensors; therefore, each sample in each time point was characterised by 16 variables. The data matrices were processed using Principal Component Analysis (PCA) for taste-masking study and Partial Least Squares (PLS) analysis for dissolution testing. All calculations and data analyses were performed in SOLO^®^ software (Eigenvector Research Inc., Wenatchee, WA, USA) and Origin (Microcal Software Inc., Northampton, MA, USA) software.

### 2.9. Statistical Analysis 

The difference between values was evaluated by the analysis of variance (ANOVA) in the Statistica 13 software (TIBCO Software Inc, Palo Alto, CA, USA). The Tukey’s test was used to check the differences among sample means for significance (*p < 0.05*). All data are presented as mean ± SD.

## 3. Results and Discussion 

The goal of this research was to formulate ODFs with adequate mechanical properties and disintegration time, containing RUP encapsulated in polymeric (EC) microparticles to provide a taste-masking effect. Different concentrations of HPMC and plasticisers were used, respectively: 8, 10, 12, 14, 16 and 18% with 30 or 50% plasticiser by the weight of the polymer. It was of great importance to ensure that the viscosity of the suspension was suitable for pouring the solutions while using an automatic applicator. It should also be taken into consideration that the viscosity of the base solution influences the subsequent stickiness in the mouth and perceived disintegration time as key attributes of ODFs with the potential to influence patient acceptability. For further research, it was decided to utilise HPMC at a concentration of 12% and glycerol at a concentration of 50% as ODFs prepared with their use were characterised by beneficial properties. The well-established safety of these excipients to be used orally was also considered [33]. The viscosity of the used solution was 1.52 Pa × s, which is in accordance with the recommendations of Woertz et al., who suggested concentrations of various cellulose derivatives (HPMC-Pharmacoat 606 and HPC-Klucel JXF 140 kDa) ranging from 7.5% to 18% for the preparation of ODF, corresponding to viscosities ranging from 0.5 to 14 Pa × s [34]. The other tested solutions had inadequate viscosity (too small or too high) and were problematic to detach from the applicator surface after drying, which was not the case at concentrations chosen for further research. The concentrations of HPMC and glycerol were optimal for placebo films and, more importantly, also for films with suspended spray-dried MPs, as it should be considered that insoluble solid particles additionally densified the solution. Furthermore, they were suitable for pouring out the films using an automatic applicator. The obtained solution was not too dense and sticky, and the suspension did not adhere to the attachment but also did not leak uncontrollably outside it; suspended particles did not quickly sediment, so they were homogeneously dispersed throughout the pouring process, which has a huge impact on the uniformity of the dose in ODFs. The addition of glycerol only in 30% *w/w* of the polymer made the films (especially with incorporated MPs) too fragile. The use of glycerol at a concentration of 50% *w/w* of polymer proved to be optimal for the developed formulations. In our previous studies, Pharmacoat606 was also utilised as a film-forming polymer and glycerol or polyethylene glycol as plasticiser. The ODFs disintegration time was mostly affected by polymer content (2.5%, 5% or 10%); as the amount of polymer was increased, prolonged disintegration time was noted. The fastest disintegration time was recorded in formulations with HPMC at a concentration of 2.5%. The lower concentration compared to the one used in this study can be explained by a different technique of film preparation, as ODFs were obtained by simple solvent casting method on Petri dish. ODFs with glycerol as a plasticiser were of higher mechanical strength in comparison to formulations with polyethylene glycol [35]. The films with polyethylene glycol were moister and tended to stick together. Interestingly, in vivo sensory tests revealed that films prepared from Pharmacoat 606 and plasticised with polyethylene glycol or a blend of glycerol and polyethylene glycol had an unpalatable taste, whereas ODFs made from glycerol alone had a more beneficial taste [35]. 

**Figure 1:**
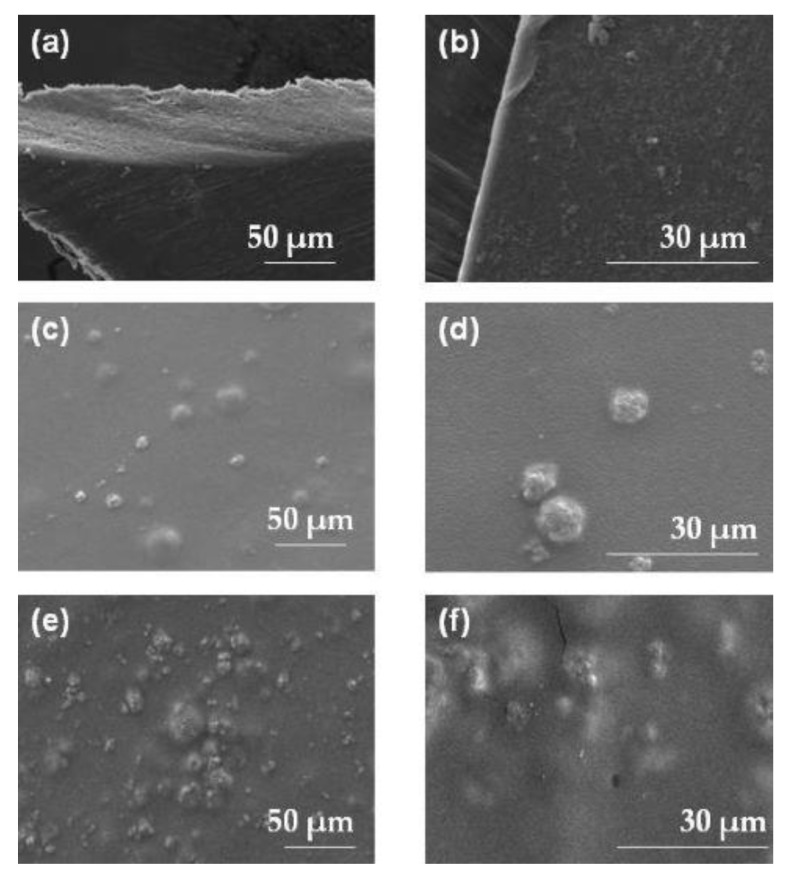
SEM picture of ODFs: placebo ODF under magnification (**a**) 2000× and 5000× (**b**); ODF with MP-S-RUP under magnification (**c**) 2000× and (**d**) 5000×; ODF with MP-A-RUP under magnification (**e**) 2000× and (**f**) 5000×.

The visual assessment showed that placebo films were smooth with shiny surfaces, while formulations containing pure RUP or MPs were of milky color and with rough surfaces due to the presence of incorporated particles. However, during the in vivo taste evaluation test, volunteers did not report any discomfort related to the presence of solid particles on the surface of ODFs. Analysis of SEM images revealed the presence of undissolved MPs (Figure 1). 

The prepared formulations were subjected to standard qualitative tests. The parameters of the films are summarised in Table 3. All formulations were comparable in terms of the thickness; however, it can be observed that with increasing the content of the solid particles, the film thickness increased. Post-hoc Tukey test revealed that formulations F2, F3 and F4 exhibited the same thickness, whereas there was a statistically significant difference between F1 and the rest of the formulations; the placebo formulation (without solid particles) (F1) was thinner. Thickness is an influential factor as it exerts an impact on overall appearance and acceptability to patients, as well as disintegration time and mechanical properties. It was stated that ODFs should have a thickness ranging from 0.05 to 0.15 mm in order to achieve rapid oral dissolution [36], which is in accordance with our results. The masses of the individual formulations were different; the addition of RUP or MPs increased the mass of a single film. All formulations were statistically different from each other in terms of weight, as confirmed by the post-hoc Tukey test. This trend in film thickness and weight can also be seen in Centkowska et al.’s work [37]. Although it was proving difficult to ensure uniform dispersion of the particles, as the process of obtaining the films consisted of several steps (mixing, casting, drying), the particles were homogeneously dispersed due to the appropriate viscosity of the solution, and the RUP content corresponded to the theoretical assumptions and was reproducible. The differences in the in vivo disintegration time measured by healthy volunteers as well as in vitro by Petri dish and drop methods were not statistically significant, which is in good agreement among each other. Most importantly, recommended disintegration time remained below 30 s, regardless of the method used. 

Residual water presence is essential for obtaining elastic films—this parameter might affect the tensile properties. A correlation exists between the mechanical properties of the film and the residual water, acting as a plasticiser. However, it should be borne in mind that high water content can result in the sticking of the films [1]. The moisture content depends on the material of the film and the method used in the production, but it is mostly within the range of 3–11%. Łyszczarz et al. obtained 2.8% of moisture content for ODFs prepared by electrospinning, 7.9% for 3D printed films and 10.9% for casted ODFs [38]. These films were prepared from an aqueous solution of polyvinyl alcohol. For formulations based on chitosan/pectin polyelectrolyte complexes it ranged from 4.9 to 7.9% [29] and from 2.2% to 4.1% for HPMC films [37]. In the case of formulations made of maltodextrin, a residual amount of water ranging between 8.0 and 11.0% was necessary to provide the films with suitable plasticity and tensile strength [39]. It is also notable that HPMC belongs to moderately hygroscopic materials (class III, based on the classification by Callahan et al.), for which the moisture content does not significantly increase after storage at relative humidities below 60% [40]. Low moisture content is favorable for the chemical and microbiological stability of the product [24,28]. F2, F3 and F4 exhibited the same moisture content, whereas there was a statistically significant difference between F1 and the rest of the formulations (Table 3).

The mechanical properties of films are influenced by various factors: the type of polymer and plasticiser used, amount of residual solvents, the thickness of single ODF, production process, as well as the type and amount of drug. In the context of ODFs, the set of mechanical characteristics is a very important parameter, as those properties provide information on the strength, robustness and ductility of the film, which is important while their packaging, transporting and handling. The only guideline given by Ph., Eur. and USP is that ODFs should possess adequate mechanical properties; there are no indications of specific values or test methods [1,4,9,11]. 

Tear resistance and tensile strength specify the stress needed for film breaking, while the percent of elongation at break determines the possible material deformation until it tears. Importantly, the films characterised by greater TS and E values tend to be hard and brittle. Elongation is a kind of deformation describing a simple change in shape under applied stress and determining the amount to which a material can be stretched before it breaks. The point at which the sample ruptures after a corresponding extension in length is defined as a percent elongation break, which is generally computed by dividing the value of the increase in length of film × 100 by the initial length of the film. The type and content of the polymer, as well as the amount of drug and plasticiser are found to have a deep influence on the percentage elongation of the ODFs. Film elongation increases with the increasing plasticiser content. For example, films made of sodium carboxymethylcellulose (NaCMC) and carbomer were found to exhibit low elasticity, whereas films made of ethyl hydroxypropylcellulose with carbomer demonstrated higher elasticity. Furthermore, an increase in the concentration of HPMC and NaCMC in the films results in a decrease in the elastic modulus [28,30].

As there are no specified limits for viscoelastic properties that correspond to good ODF performance during manipulation, the reference should be made to the experimental literature data. Thus, films that are too flexible can be malleable, e.g., during removal from packaging. Brittle films, on the other hand, can cause cracks during manufacturing and cutting. Young’s modulus (E) is a measure of the rigidity or stiffness of a material. The greater the modulus, the stiffer the material—an idealised rigid body would have an infinite value of E. It is assumed that an ideal ODF should be characterised by moderate TS and low E values, as well as a high percentage of elongation [1,28,30,41]. It should also be kept in mind that the solids content affects the mechanical properties of the films; the more solids are in the film, the more fragile and less flexible the films become, which could be explained by the fact that the solid particles disturb the matrix continuity [28,30,34]. The properties of the obtained ODFs are shown in Table 4. They were of rigid structure (mechanically robust, with high E values) but did not crumble. As expected, the E values increased with increasing particle content, which affected the stiffness of the ODFs. Such a trend can also be seen in Brniak et al. [42]. TR and TS parameter was rather low (highest values were recorded for placebo films, 18.35 ± 0.27 and 7.12 ± 0.06 (N/mm^2^), respectively), indicating that the films were characterised by slight resistance to tearing, although our results for TS were comparable to those obtained by Potaś et al. [29]. The placebo films were characterised by the highest values of TR, TS and EB, but the lowest of E (for the other formulations, increased E with increasing solid particles content (RUP< MP-S-RUP < MP-A-RUP) was observed). The formulations containing solid particles had considerably lower folding resistance compared to placebo (Table 4). According to the obtained statistical data, samples were significantly different (*p < 0.05*). Post-hoc Tukey test revealed that formulation F4 was significantly different from all other formulations in terms of TR. Similar values were observed for F1 and F3 and also for F2 and F3. The test also revealed that considering tensile strength, F1 and F4 were significantly different from other formulations. There was no significant difference between F2 and F3. EB was low with a maximum of 2.74 (%) for placebo films.

The mechanical features of ODFs can also be dependent on the water content; however, as it was shown in Table 3, the water content was in small amounts, which should not affect the mechanical properties and which is also important to reduce the potential growth of microorganisms. In comparison, the films obtained by Centkowska et al. were characterised by higher values of each parameter and were more resistant to folding [37], whereas those developed by Brniak et al. were comparable with regard to TR and EB [42]. 

**Figure 2:**
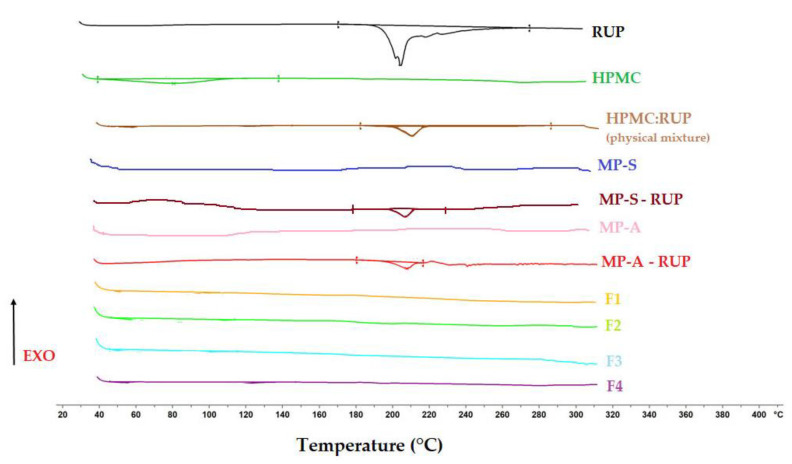
DSC thermograms of RUP; HPMC; physical mixture of RUP and HPMC; MPs placebo, MP-S-RUP and MP-A-RUP; and films F1, F2, F3 and F4.

In order to evaluate physical drug properties and potential incompatibilities with other components, DSC was applied. RUP, HPMC and their physical mixtures; MPs placebo, MP-S-RUP and MP-A-RUP; and ODFs F1, F2, F3 and F4, were assessed (Figure 2). The thermogram of pure RUP presents an endothermic event at 200.35 °C characterised by a sharp peak corresponding to its melting point [43,44]. Thermograms of MPs placebo show that there are no thermal events that indicate that used aqueous dispersion of EC is in an amorphous state [45]. Converting RUP into microparticles formed by spray drying did not change the solid-state nature of the drug. Minor changes in its melting point occurred in MP-S–RUP and MP-A-RUP, which is probably due to the fact that excipients used can slightly change the physicochemical properties of a drug during spray drying [46]. In the physical mixture of HPMC and RUP, there is a peak corresponding to the RUP melting point. Thus, this peak would be expected in the case of the retention of the RUP crystalline state in the films. However, in DSC curves for the films, F2, F3 and F4, no melting peak of RUP was detected, probably as RUP was an undetectable concentration or its crystallinity was reduced as a result of thermally induced interactions between RUP and HPMC, which might point out high the miscibility of both compounds.

Palatability is of great importance to determine the drug acceptability for patients. It is valid to assess taste early on during development. Many taste-masking strategies are available, among others emulsification/solvent evaporation, centrifugal extrusion, coacervation, spray drying or supercritical carbon dioxide (scCO2) assisted technique, which main advantage lies in the possibility of achieving a high separation of the supercritical solvent and the processed products, involved drug particle size reduction or the modification of some of the physicochemical and structural properties, especially of poorly water-soluble drugs [47,48,49,50]. While utilising the spray drying technique, the bitter drug is dissolved or dispersed along with the polymer in a suitable solvent and then subjected to spray drying. The dried product contains particles incorporating taste- and flavor-masked drugs. This method provides an alternative route to taste-masking by providing a physical barrier coating [16]. A pleasant mouth feeling, rapid disintegration and acceptable taste are crucial features of drug dosage forms. The human taste panel continues to be the unquestioned gold standard in taste assessment [3,8]. In order to evaluate taste, a consumer preference panel is created, using healthy adult volunteers trained in “sip and spit” tasting techniques [47]. However, taste evaluation is challenging, especially in pediatrics, for safety and ethical reasons. Therefore, several in vitro taste evaluation methods were developed to overcome these obstacles, and these include an electronic tongue (ET) utilisation [51,52]. ET sensors can be used to mimic human taste perception across the five major taste categories (bitter, salty, sour, sweet, and umami). The taste patterns generated are then used to determine palatability. An important advantage of chemical sensors is their selectivity, i.e., the ability to measure the concentration of a specific component in the presence of other components of a complex sample, resulting from the presence of a chemically selective layer responsible for the process of recognition of the analyte. In a sensor array, several potentiometric electrodes are usually applied, in which synthetic layers—polymer membranes with electroactive additives—imitate receptor layers in cell walls of taste buds. The signals coming from the chemical sensor array are recorded in the form of a data matrix. Each sample is represented by a unique and characteristic image vector—a point in the multidimensional data space, which is a unique "fingerprint", a "chemical image", characteristic for a given sample. Samples with similar chemical images are characterised by high similarity; they have similar compositions or properties. Multidimensional measurement data from a sensor array are difficult to analyse and visualise. In order to reduce the dimensionality of the problem and eliminate the repetitive information, appropriate mathematical procedures are applied, e.g., Principal Components Analysis (PCA). The basis of PCA is to analyse the distribution of variation in data from multivariate measurements and then find new directions in space, orthogonal to each other, that maximise the display of this variation. Representation of data on a two- or three-dimensional graph leads to clusters of objects with similar properties (usually, the first two principal components allow for appropriate differentiation of samples) [50,51,52,53].

Evaluation of taste-masking efficiency was conducted by three independent methods: in vivo in human taste panel by six healthy volunteers, ET and in vitro drug dissolution. Sensory evaluation of ODFs was scored as follows: 0—not bitter, 1—slightly bitter, 2—moderately bitter, 3—very bitter. All probands assessed formulation F3 as slightly bitter and F4 as non-bitter or slightly bitter in comparison to that with pure RUP (F2), which was determined as very bitter (Table 5).

**Figure 3:**
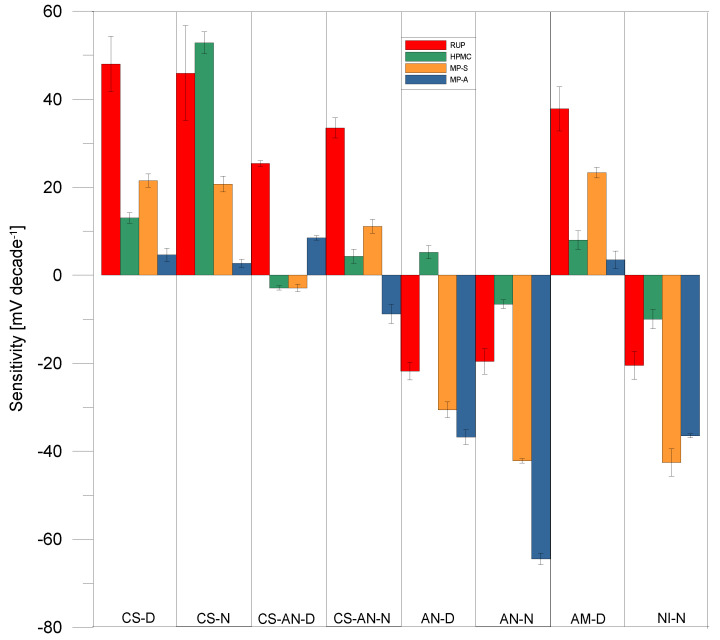
Sensitivity of electrodes towards RUP, HPMC, MP-S, MP-A.

Construction of a highly discriminative electronic tongue demands the fabrication of sensors with a differentiated selectivity and, at the same time, satisfactory sensitivity towards the components of the studied samples. For this purpose, eight different electrodes with different compositions (plasticisers, active substances) of polymer membrane were constructed (Table 2). The first important part of the research was to check the sensitivity of the achieved electrodes to RUP. Thus the calibration curves of electrodes in RUP of concentration 10^−6^–10^−3^ mol/L were determined. As can be seen (Table 6), part of the sensors exhibited cationic function and the other anionic response of various sensitivity (from few mV/dec to Nernstian value). Then the second important part of the research was testing the sensitivity of the obtained electrodes towards HPMC, MP-S-RUP and MP-A-RUP, as presented in Figure 3. Depending on the active substance in the membrane, the electrodes also showed a cationic or anionic response to individual excipients, as in the case of RUP. Diversified, high, varied sensitivity of the electrodes proved the possibility of their application to a potentiometric sensor array used coupled with chemometrics for the analysis of pharmaceutical samples containing RUP.

**Figure 4:**
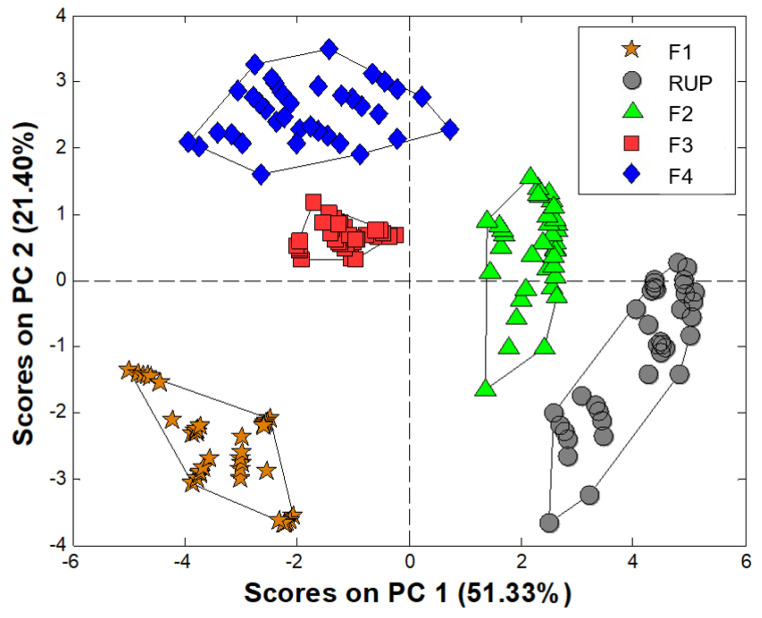
PCA score plot of electronic tongue results for the studied formulations (F1, F2, F3, F4) and RUP.

The prepared sensor array of ET was applied to check the taste-masking efficiency of the studied formulations. Such test demands comparison with a bitter standard (as such, pure drug most often is applied) and placebo. Thus, five types of samples were tested according to the procedure described in the experimental section. The responses of every sensor were given as potentiometric signals in a function of time. Taste evaluation was performed for signals recorded after one minute from the start of the release, and the resulting PCA score plot is presented in Figure 4. 

**Figure 5:**
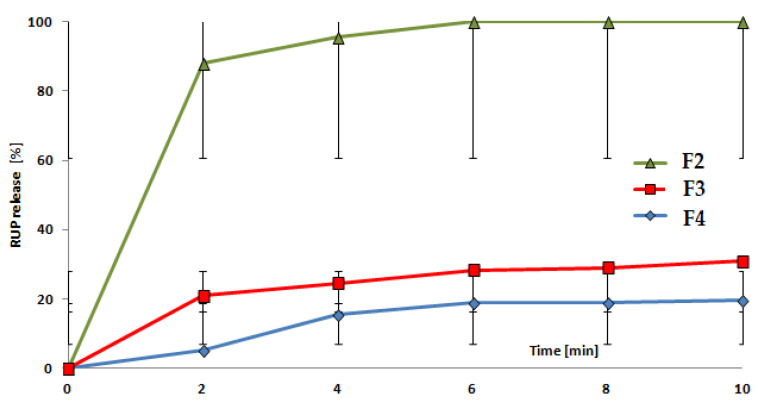
In vitro RUP release from ODFs: F2, F3, F4 conducted in paddle apparatus.

All samples formed distinct clusters that are easily separable from each other. On the opposite sides of the plot, placebo and pure RUP are visible. They are characterised by the lowest (placebo) and highest (pure drug) values of the first Principal Component, which captures most of the variance of the data set. The studied formulations F2, F3 and F4 are placed between placebo (F1) and pure RUP, showing moderate taste between the two, which is correct and was expected. The clusters of formulations are not overlapping, and they are placed in a sequence F2–F3–F4, which shows an increase in bitterness (F4 is the closest to placebo, F2 is the closest to RUP). It correlates well with both the human taste panel (Table 5) and dissolution test results (Figure 5), where the effect of introducing the drug in the form of microparticles led to the attenuation of the sensed bitterness. Moreover, it can be observed that the best taste-masking was achieved in the F4 formulation, where Aquacoat^®^ ECD was applied for the formation of microparticles. It is also in perfect agreement with results obtained by the human panel and pharmacopoeial dissolution tests. We assumed that the differences in the degree of taste-masking efficiency between formulations obtained using MP-S-RUP or MP-A-RUP were probably affected by various, additional excipients present in the aqueous dispersions of ethylcellulose used in our experiment, notably oleic acid and fractionated coconut oil in Surelease^®^, cetyl alcohol and sodium lauryl sulfate in Aquacoat^®^ ECD. The shape of obtained microparticles in both cases was similar—they were smooth and spherical [17]. Perhaps, the applied composition of the aqueous dispersions allows faster/slower diffusion of water into the particles. 

Signals of electrodes forming the sensor array of the electronic tongue were recorded during 10 min of formulation release. Due to the cross-sensitivity of sensors, these outputs are related to the growing concentration of RUP, but they were also strongly influenced by excipients that are also released (Figure 3). Thus, a supervised data analysis technique to extract information of RUP release from sensor responses was applied.

**Figure 6:**
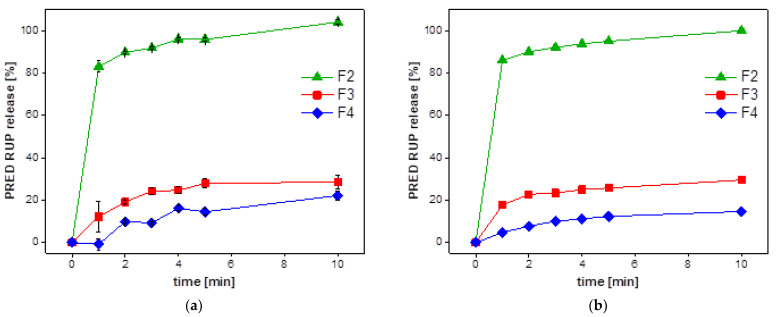
Electronic tongue prediction of RUP release: (**a**) results for the train set—points of dissolution curve presented as mean ± SD, *n* = 5; (**b**) results for independent test set, *n* = 1.

Partial Least Squares Regression (PLS) is a technique that combines features from PCA and multiple regression for the prediction of the dependent variable(s) from a set of independent variables. The original data (electronic tongue outputs in this case) are transformed into a set of a few intermediate linear latent components that are useful for predicting the dependent variable(s) [54]. This data analysis technique was applied many times for various identification, recognition, discrimination and quantification tasks performed by electronic tongue in our lab [55,56,57]. It was also found to be very useful for the prediction of drug release from various formulations, the results of which we published recently [46]. PLS model developed in this work was based on electronic tongue signals in appropriate time points as X data, whereas Y data, i.e., target matrix, was constructed based on values of %RUP release that were determined by pharmacopoeial dissolution test. Five replicates of each studied formulation, F2, F3 and F4, were used for calibration as a training set, whereas the remaining replicate number six served as a validation sample (independent test set). After the model development, the electronic tongue system was capable of predicting the amount of RUP that was released from the respective formulation based on electrodes’ signals. The values of RUP release for a few timepoints formed respective dissolution curves for the studied three formulations, both for the training set and the testing samples (Figure 6a,b, respectively). It must be underlined that the values predicted by the electronic tongue system are estimates; they do not provide accurate values of RUP release. Nevertheless, they exhibit very high similarity to the results of dissolution tests presented in Figure 4. Moreover, the outputs of the PLS model show general tendencies discerning the studied formulations according to release dynamics. According to electronic tongue signals, F2 released RUP very fast, whereas F3 and F4 were characterised by much slower dynamics of RUP release. The slowest release was observed for F4, where Aquacoat^®^ECD was applied to form microparticles. The results for the train set and test set were in perfect agreement, and they also correlated perfectly with the picture given by the standard dissolution test. They also additionally confirm the results presented above for taste-masking examined by electronic tongue system. 

## 4. Conclusions

Currently, when developing new medicinal products, it is expected that not only patient outcomes will be considered, but attention will also be paid to the overall patient experience. It is known that convenience is increased with ODFs across all patient populations. Moreover, the efficiency of the taste-masking effect is often a key determinant for the success of specialised dosage forms. ODFs with RUP in the form of ethylcellulose microparticles were successfully designed to reduce the perception of the bitter taste. ODFs were obtained by a simple, fast and reproducible method using an automatic applicator. Excipients of well-established safety (HPMC, glycerol) were used to design the formulation. Due to the necessity to suspend the MPs in the casting solution, it was a technological challenge to achieve homogenous drug content per dosage unit and sufficient mechanical properties for film handling. Although the process of obtaining the films consisted of several steps (mixing, pouring, drying), the particles were homogeneously dispersed, and each film of the desired size contained the proper dose of the drug. The incorporation of MPs affected the mechanical features of the films by weakening their robustness, but they remain sufficient for handling. The taste-masking effect was maintained as well, as confirmed by three independent methods. In the case of ET, all samples formed distinct clusters that were easily separable from each other, which indicated differences in taste patterns among formulations. The clusters of formulations were placed in a sequence F2–F3–F4, which showed bitterness (F4 is the closest to F1—placebo film, F2 is the closest to pure RUP). It correlated with dissolution test results, where the effect of introducing RUP in the form of microparticles led to the attenuation of the sensed bitterness. F2 released RUP very fast, whereas F3 and F4 were characterised by much slower dynamics of RUP release. It was also in agreement with results obtained in the human panel. Although undissolved drug particles or ethylcellulose MPs protruded above the surface of the film after drying, this did not cause unpleasant sensations, i.e., graininess or sandiness, according to the volunteers.

## Figures and Tables

**Table 1:** Composition of casting solutions poured on the surface of the applicator.

Ingredient (%)	Formulation			
	F1	F2	F3	F4
**RUP**	-	0.25	-	-
**MP-S-RUP**	-	-	0.51	-
**MP-A-RUP**	-	-	-	0.62
**HPMC**	12.0	12.0	12.0	12.0
**Glycerol**	6.0	6.0	6.0	6.0
**Purifiedwater**	82.0	81.75	81.49	81.38

**Table 2:** Composition of sensor array of the electronic tongue.

El. no.	Electrode Type	Ionophore (% *w/w*)	Lipophilic Salt (% *w/w*)	Plasticiser (% *w/w*)	Polymer (% *w/w*)
1–2	CS-D	-	KTpCPB (3%)	DOS (65%)	PVC (32%)
3–4	CS-N	-	KTpCPB (3%)	o-NPOE (65%)	PVC (32%)
5–6	CS-AN-D	-	TDMA-TCPB (3%)	DOS (65%)	PVC (32%)
7–8	CS-AN-N	-	TDMA-TCPB (3%)	o-NPOE (65%)	PVC (32%)
9–10	AN-D		TDAC (3.5%)	DOS (64%)	PVC (32.5%)
11–12	AN- N	-	TDAC (3.5%)	o-NPOE (64%)	PVC (32.5%)
13−14	AM-D	amine ionophore I (5%)	-	DOS (68%)	PVC (27%)
15–16	NI-N	-	TDMAN (6%)	o-NPOE (62%)	PVC (32%)

**Table 3:** Physicochemical characteristic of prepared ODFs (*n* = 3).

Formulation	Thickness [µm]	Weight [mg]	Moisture Content [%]	RUP Content [%]	Disintegration Time [s]		
					In Vivo	Petri Dish	Drop Method
**F1**	131.03 ± 0.82 ^a^	16.05 ± 0.13 ^a^	6.21 ± 0.47 ^a^	not applicable	18.00 ± 0.82 ^a^	17.21 ± 0.19 ^a^	22.50 ± 0.58 ^a^
**F2**	133.21 ± 0.49 ^b^	18.03 ± 0.13 ^b^	3.74 ± 0.29 ^b^	99.23 ± 2.55 ^a^	18.75 ± 0.50 ^ac^	18.34 ± 0.26 ^b^	24.00 ± 0.82 ^b^
**F3**	133.92 ± 1.27 ^b^	20.03 ± 0.17 ^c^	3.92 ± 0.18 ^b^	93.26 ± 1.72 ^b^	20.25 ± 0.96 ^bc^	19.18 ± 0.13 ^c^	24.75 ± 0.50 ^b^
**F4**	134.91 ± 0.82 ^b^	21.43 ± 0.46 ^d^	3.55 ± 0.37 ^b^	101.29 ± 2.26 ^a^	21.25 ± 0.96 ^bd^	20.13 ± 0.18 ^d^	25.0 ± 0.82 ^b^

Note: Different letters (a, b, c, d) in the same column indicate the significant differences (*p* < 0.05).

**Table 4:** Physicomechanical properties of films (*n* = 3).

Formulation	Tear Resistance [N]	Tensile Strength [N/mm^2^]	Elongation at Break [%]	Young’s Modulus [MPa]	Folding Endurance
**F1**	18.35 ± 0.27 ^a^	7.12 ± 0.06 ^a^	2.74 ± 0.03 ^a^	611.1.1 ± 0.45 ^a^	>300
**F2**	17.31 ± 0.30 ^bc^	6.41 ± 0.10 ^b^	1.52 ± 0.04 ^b^	792.3 ± 0.73 ^b^	≤100
**F3**	17.86 ± 0.33 ^ac^	6.61 ± 0.05 ^b^	2.18 ± 0.06 ^c^	692.4 ± 0.66 ^c^	≤100
**F4**	15.49 ± 0.42 ^d^	5.94 ± 0.15 ^c^	1.37 ± 0.04 ^d^	966.3 ± 0.19 ^d^	≤100

Note: Different letters (a, b, c, d) in the same column indicate the significant differences (*p* < 0.05).

**Table 5:** Sensory evaluation of designed ODFs: 0—no bitterness, 1—slight bitterness, 2—moderate bitterness, 3—significant bitterness.

Volunteer/ Formulation	Score		
	F2	F3	F4
A	3	1	1
B	3	2	1
C	3	0	0
D	3	1	1
E	3	1	0
F	3	1	0

**Table 6:** Potentiometric working characteristic for 8 electrodes.

Lp	Electrode Type	Sensitivity ± s, mV/Decade	Linear Range −log c, mol/L	R ± SD
1	CS-D	4.1 ± 6.3	3–5	0.9980 ± 0.0035
2	CS-N	53.5 ± 10.8	3–5	0.9997 ± 0.0003
3	CS-AN-D	24.8 ± 0.6	3–6	0.9884 ± 0.0034
4	CS-AN-N	31.4 ± 2.3	3–6	0.9720 ± 0.0090
5	AN-D	−19.5 ± 2.0	4–6	0.9897 ± 0.0150
6	AN-N	−22.4 ± 2.9	4–6	0.9919 ± 0.0063
7	AM-D	40.4 ± 5.0	3–5	0.9981 ± 0.0015
**8**	NI-N	−22.8 ± 3.2	4–6	0.9936 ± 0.0079

## Data Availability

Data are contained within the article; raw data are available upon request.
